# Donor‐specific HLA antibodies after fresh decellularized vs cryopreserved native allograft implantation

**DOI:** 10.1111/tan.14077

**Published:** 2020-10-13

**Authors:** Iuliana Coti, Sabine Wenda, Alexandra Andreeva, Alfred Kocher, Guenther Laufer, Gottfried Fischer, Martin Andreas

**Affiliations:** ^1^ Department of Surgery, Division of Cardiac Surgery Medical University of Vienna Vienna Austria; ^2^ Department of Blood Group Serology and Transfusion Medicine Medical University of Vienna Vienna Austria

**Keywords:** cryopreserved native allografts, donor‐specific HLA antibodies, fresh decellularized allografts

## Abstract

This study aims to compare the immunogenicity of fresh decellularized with cryopreserved native heart valve allografts to identify potential immunological risks in subsequent organ transplantations. We measured the induction of allogeneic HLA class I and II specific antibodies and characterized donor‐specific antibodies by Luminex‐based single beads assay in both groups. Serum samples were collected before valve replacement, at 3 and 24 months postoperatively. Donor‐specific HLA antibodies were assessed positive if the mean fluorescent intensity (MFI) was >1000. Between November 2016 and April 2017 patients with fresh decellularized homografts (n = 4) and cryopreserved native homografts (n = 4) were analyzed. Patients receiving cryopreserved native allografts reacted with broad HLA‐specific antibody response. Antibodies were directed against mismatched HLA antigens of the donors but also against HLA specificities not present on the homograft with many antibodies having mean fluorescence intensity values >10 000. While HLA class I specific antibodies showed a significant increase (*P* = .002) in their MFI values on day 90, HLA class II specific antibodies did not show a significant increase (*P* = .069). In the fresh decellularized homografts group, no significant antibody induction was observed. Consequently, the native group presented significantly higher MFIs for HLA antibodies on day 90 compared with the patients receiving decellularized allografts (*P* = .021). No detectable HLA antibody response was observed after implantation of decellularized in comparison with cryopreserved native allografts. Lower immunogenicity as compared with native homografts might increase the chance of receiving a transplant if will be required later in the life of the patients.

## 1 INTRODUCTION

Aortic or pulmonary valve replacement or a Ross procedure with human homografts may be required because of the lack of alternatives in children and young adult patients. The use of cryopreserved human allografts offered better long‐term results in terms of valve hemodynamics and long‐term freedom of reoperation, as well as lower morbidity and mortality in comparison with xenografts or mechanical prostheses.^1^, ^2^ Excellent results were achieved after the Ross operation with good freedom from reoperation and excellent hemodynamics at long‐term follow‐up.^3^ However, the majority of patients implanted with cryopreserved homografts develop antibodies against HLA, specific for the transplanted tissue.^4^, ^5^ Considering that the main target patient population undergoing valve replacement with human allografts are children or young adults, in whom later heart transplantation may be necessary, a significant humoral response against valve tissue is a matter of concern. The use of decellularized human valve allografts during the last decade intended to overcome this limitation, avoiding a vigorous immune response. They showed better outcomes regarding freedom from explantation and structural valve degeneration, when compared with bovine jugular vein conduits or cryopreserved human homografts.^6^ In order to evaluate the superiority of fresh decellularized allografts to cryopreserved native grafts regarding the development of humoral antibodies against donor allografts, the presence of anti‐HLA antibodies class I (HLA‐A, B, C) as well as anti‐HLA antibodies class II (HLA‐DR, DQ, DP) was analyzed using the Luminex Single Antigen bead assay in patients implanted either with a fresh decellularized or with a cryopreserved native homograft in the pulmonary or aortic position.

## 2 MATERIALS AND METHODS

### 2.1 Study design

Between November 2016 and April 2017, we prospectively enrolled eight patients implanted with either a pulmonary or an aortic homograft at our institution. These patients were preoperatively included in our institutional registries with postoperative clinical follow‐ups including blood tests, ECG, and echocardiography after pulmonary (1362/2016) or aortic homograft implantation (2201/2016). The Institutional Review Board approved these registries and patients signed the informed consent preoperatively. The patients selected for the HLA analysis were operated into this time frame and gave consent for this additional analysis. We analyzed HLA specific antibodies for HLA‐class I (A, B, C) and class II (DR, DQ, DP) by Luminex Single Antigen bead assays in two groups: four patients after fresh decellularized homograft implantation and four patients after cryopreserved native homograft implantation. Serum samples were collected before valve replacement, at 3 and 24 months postoperatively. Donor specific HLA antibodies (DSA) were assessed positive at a mean fluorescent intensity (MFI) higher than MFI > 1000.

### 2.2 Surgical techniques

The operations were performed through a median sternotomy with cardiopulmonary bypass and mild hypothermia (32°C‐34°C) with intermittent antegrade and retrograde cold blood cardioplegia. In case of the Ross procedure, the pulmonary homografts were implanted using running sutures both proximal and distal, and the autografts as a root replacement with running sutures proximal and distal and reinsertion of the coronary buttons. Three patients received an isolated aortic root replacement with an aortic homograft, all of them being fresh decellularized. No extension of the homografts with pericardial patches was performed in any of the cases.

### 2.3 Study endpoints

The primary endpoints were the assessment of the immunogenicity after aortic or pulmonary valve replacement with either a fresh decellularized or a cryopreserved native homograft as described above. As secondary endpoints, we assessed the valve performance by a clinical follow‐up including a transthoracic echocardiographic assessment.

### 2.4 Homograft procurement and processing

The cryopreserved homografts were procured from a European tissue bank after being processed from Bislife, Leiden, The Netherlands. The fresh decellularized homografts were procured and prepared under sterile conditions from living donors requiring heart transplantation at our institution and shipped on ice in the antibiotic solution for decellularization to Corlife, Hannover, Germany. The decellularization process implies the use of different detergents as 0.5% sodium deoxycholate and 0.5% sodium‐dodecylsulfate for 36 hours at room temperature. Homografts are then washed with NaCl 0.9% solution and stored at 2°C to 8°C (up to 3 weeks) until implantation. This process does not imply freezing/cryopreservation.

### 2.5 HLA typing of valve donor

Donors of the fresh decellularized homografts were already HLA typed at our institute when waiting for heart transplantation. HLA typing was performed using the HISTO SPOTSSO assay (BAG, Lich, Germany) according to the manufacturer's instructions. For donors of cryopreserved homografts no HLA typing was initially available. Small pieces of the valve or the surrounding muscle tissue were homogenized using a homogenisator (SpeedMill PLUS, Jena Analytik, Jena, Germany). Subsequent DNA extraction was performed in a Maxwell 16 Instrument (Promega, Madison, Wisconsin). Whole gene typing was performed by next‐generation sequencing (NGS) for HLA‐A, B, C, DRB1, DRB3/4/5, DQA1, DQB1, and DPB1.^7^

### 2.6 HLA antibody analysis

Antibody detection for HLA class I and class II was performed using the Luminex Single Antigen bead assay (LABScreen Single Antigen, Thermo Fisher Scientific Inc., Waltham, Massachusetts) according to the manufacturer's instructions. All sera were pretreated by heat inactivation. Results were analyzed in the HLA Fusion software (Thermo Fisher Scientific Inc.). Antibody tracking was also performed in the HLA Fusion software.

### 2.7 Virtual panel reactive antibodies

HLA specific antibodies with an MFI > 1000 were included in calculations for virtual panel reactive antibodies. These antibodies have a negative impact on further heart transplantations, they are a contraindication for allocation. The vPRAs have been calculated with the Eurotransplant vPRA calculator (ETRL HLA database 2.0).

### 2.8 Statistical analysis

Descriptive statistical methods were applied to depict the study population regarding preoperative or intraoperative characteristics and postoperative outcomes. Continuous variables were presented as mean and SD or median (25th‐75th interval) for variables with a skewed distribution. The normal distribution of variables was assessed with the Kolmogorov‐Smirnov test. Total numbers and proportions were reported for categorical outcomes. Statistical calculations comparing continuous variables were made using the dependent *t* test for comparisons of groups before and after transplantation. Independent *t* test and Mann‐Whitney‐U test was applied for comparisons of the two groups defined by the homografts. IBM SPSS Statistics 26 (IBM Corp. Released 2019. IBM SPSS Statistics for Win, Version 26.0. Armonk, NY: IBM Corp.) was used for statistical analysis. A *P*‐value of less than .05 was considered as significant.

## 3 RESULTS

### 3.1 Baseline characteristics

Between November 2016 and April 2017, eight patients implanted with either a fresh decellularized or a cryopreserved native homograft in pulmonary or aortic position at our institution were analyzed (four patients fresh decellularized and the other four cryopreserved native homograft implantation). The mean age was 45 ± 14.5 years (range from 24 to 62 years) and 7 (88%) were men. At baseline, three patients had severe aortic insufficiency, another two severe aortic stenosis, and the remaining three patients combined stenosis/insufficiency of different degrees. Other baseline and perioperative details are described in Table 1.

**TABLE 1:** Patient characteristics

Factor	Cryopreserved (N = 4)	Fresh decellularized (N = 4)
Age, y, (SD) years	51 (9)	39 (18)
Gender, female (%)	0	1 (25%)
Height (SD) cm	175 (7)	176 (8)
Weight (SD) kg	79 (8)	73 (8)
Body mass index (SD) kg/m^2^	25 (3)	24 (1)
History of blood transfusions (%)	0	0
Perioperative blood transfusions (%)	2 (50)	1 (25)
Pregnancies	–	0
Previous cardiac surgery	0	0

*Note:* Continuous data are presented as the mean and SD; categorical data as total number and percentage.

### 3.2 Procedural characteristics

All patients had a bicuspid aortic valve. Five patients underwent a ROSS procedure (one with a decellularized pulmonary allograft and four with a cryopreserved graft) and three patients received an isolated aortic homograft (in all cases a fresh decellularized graft). The mean implanted homograft diameter in pulmonary position was 26.4 ± 2.2 mm and 27.3 ± 4.6 mm in aortic position. The procedural success rate was 100%, intraoperative adverse events related with the procedure occurred in two cases when a second aortic clamp time was necessary, both cases after aortic homograft implantation; in one case a tear and bleeding at distal anastomosis because of insufficient adventitial tissue occurred after removing the cross‐clamp and native pericardial tissue was successfully used to reinforce the anastomosis at the bleeding site; in the second case a mild valvular aortic insufficiency of the implanted homograft was observed at the time of weaning from CPB—in this case the distal anastomosis was partially opened and the aortic valve carefully inspected; no distortion of the root geometry or possible leaflets defects from suturing were observed and the distal anastomosis was readapted. Concomitant procedures, excluding the patients in whom a Ross operation was performed, were conducted only in one case (atrial septal defect closing). Mean overall cardiopulmonary bypass (CPB) and cross‐clamp times (XCT) were 179.6 ± 33.1 and 141.9 ± 29.9 minutes. The early and intermediate‐term survival rate was 100%.

### 3.3 Generation of HLA‐specific antibodies

**FIGURE 1:**
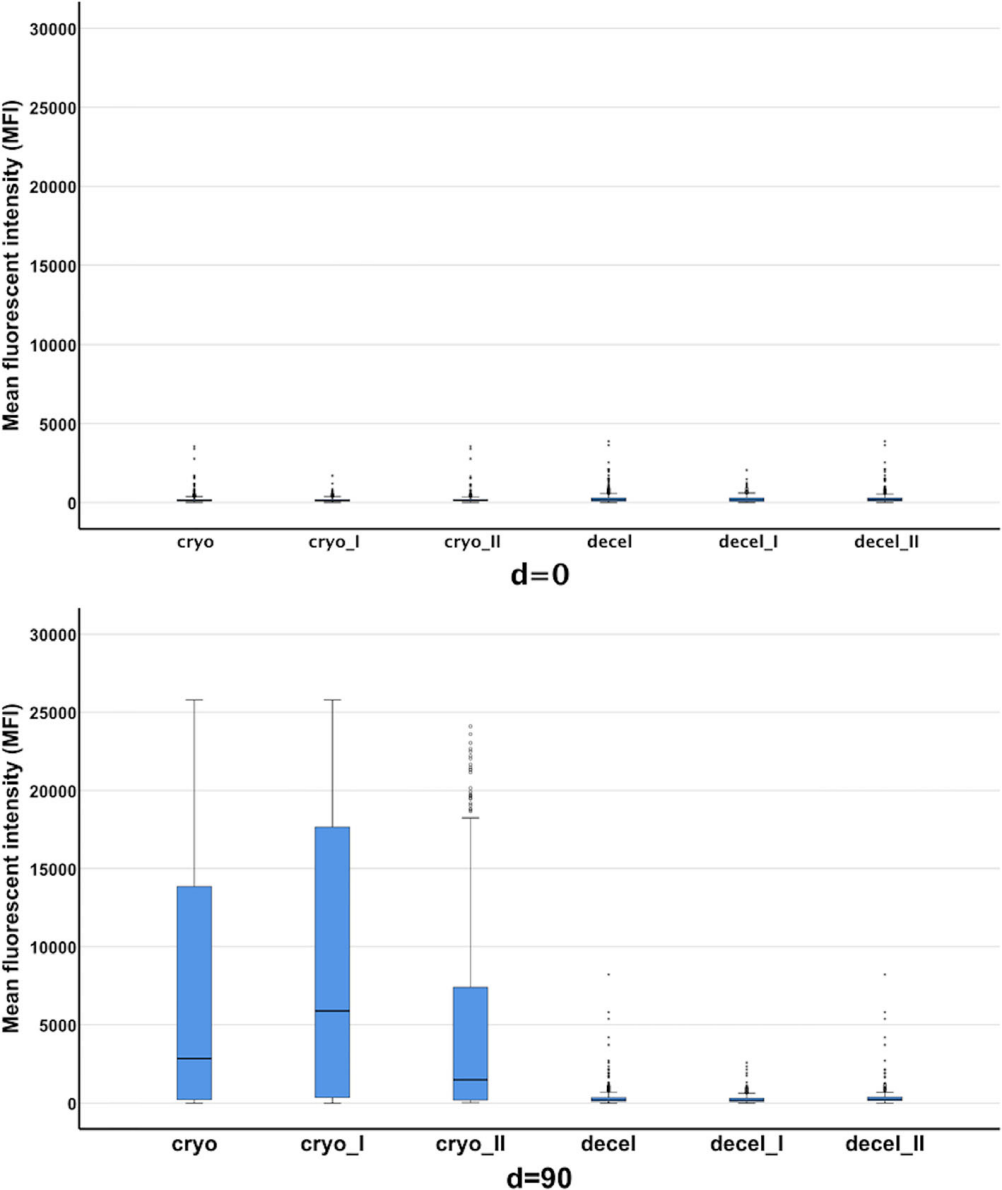
Legend: Median MFIs on d = 0 and d = 90 grouped by homografts (native cryopreserved—“cryo” and fresh decellularized—“decel”). The black bold line in the boxplots denotes the median MFI value for pre‐homograft implantation samples; data show either MFIs of HLA class I and class II (“cryo,” “decel”) or MFIs of HLA class I (“cryo_I,” “decel_I”) and of HLA class II (“cryo_II,” “decel_II”)

Statistical analyses have been performed by comparing the sum of all MFIs of a test (either taking into account all MFIs or separated according to HLA class I or HLA class II specificities) in patients. For each specificity one bead was taken into account; in case the allele of the donor was known, the allele‐specific bead was used; in case of generic typing of the donor, the bead of the respective serological group with the highest MFI value was used for statistical analyses. Patients have been grouped according to the homograft they received (cryopreserved or decellularized) and according to time after implantation (d = 0 or d = 90). The distribution of MFI values pre‐ (d = 0) and post‐homograft implantation t (d = 90) is shown in Figure 1 for both groups. Additionally, MFI values for class I and class II are shown separately.

Comparison between patient groups on day = 0 (Figure 1A) did not show any significant difference (*P* = .564). However, 90 days after implantation the group of patients having received the cryopreserved allografts showed significantly higher sums of MFIs (*P* = .021) as compared with the patients receiving decellularized allografts (Figure 1B). This indicates that a broad immunization against allogeneic HLA antigens has taken place in the former patient group while patients having received decellularized antibodies showed no or minimal immunization only.

If comparisons are made between pre and post samples, the difference between d = 0 and d = 90 was significant only in the patients receiving cryopreserved homografts (*P* < .001).

Supplemental Table 1 shows the sum of MFI values of all HLA specific antibodies for each patient divided into HLA class I and class II specific antibodies for d = 0 and d = 90.

When the analyses were refined to consider HLA class I and HLA class II specific antibodies separately, the patients receiving cryopreserved homografts showed only time‐dependent significant increases for class I specific (*P* = .002) but not for HLA class II specific antibodies (*P* = .069). As expected also in these analyses the group of patients receiving decellularized homografts showed no significant increase of antibodies (HLA class I *P* = .368, HLA class II *P* = .308).

The wide immunization is indicated by the mean MFI values grouped by homografts and was determined at d = 0 and d = 90 (Table 2).

**TABLE 2:** Mean MFI values of HLA specific antibodies

	Mean MFI (SD)		
	d = 0	d = 90	*P* value
Cryo	193.9 (10.8)	7160.1 (314.1)	.001
Cryo I	166.1 (8.0)	8719.7 (437.5)	.002
Cryo II	232.9 (23.1)	4967.5 (404.7)	.069
Decel	241.8 (13.2)	331.9 (22.1)	.327
Decel I	205.2 (10.9)	263.0 (15.4)	.368
Decel II	293.2 (27.6)	428.6 (48.0)	.308

*Note:* the mean MFIs for patient groups receiving different homografts (cryopreserved—Cryo and decellularized—Decel) both pre‐ (d = 0) and posttransplant (d = 90). Total values and a separate analysis of HLA class I and class II for both cryopreserved (Cryo I, Cryo II) and decellularized (Decel I, Decel II) homografts were provided, and the *P*‐value obtained by comparing the baseline values (d = 0) with the values at 3‐months (d = 90) in for all groups.

HLA specific antibodies with an MFI > 1000 are included in calculations for virtual panel reactive antibodies. Virtual PRAs were absent in the decellularized group and above 95% in all patients implanted with a native cryopreserved homograft (*P* < .001). The development of this vPRA is shown in Table 3 for both groups pre‐ and post‐homograft implantation.

**TABLE 3:** Virtual panel reactive antibodies

	vPRA in percent *P* value		
	Type of homograft	d = 0	d = 90
pat_1	cryopreserved	0%	95,6%
pat_2	cryopreserved	0%	99,6%
pat_3	cryopreserved	0%	98,57%
pat_4	cryopreserved	0%	95,01% *P* < .001
pat_1	decellularized	0%	0%
pat_2	decellularized	0%	0%
pat_3	decellularized	0%	0%
pat_4	decellularized	0%	0%

*Note:* For each of the patients the pre‐ and posttransplant panel reactivity, that is, the expected frequency of donors within the Eurotransplant area that would have a positive virtual crossmatch, vPRAs have been calculated with the Eurotransplant vPRA calculator (ETRL HLA database 2.0). The *P* value designates the development of PRA between the two groups.

**FIGURE 2:**
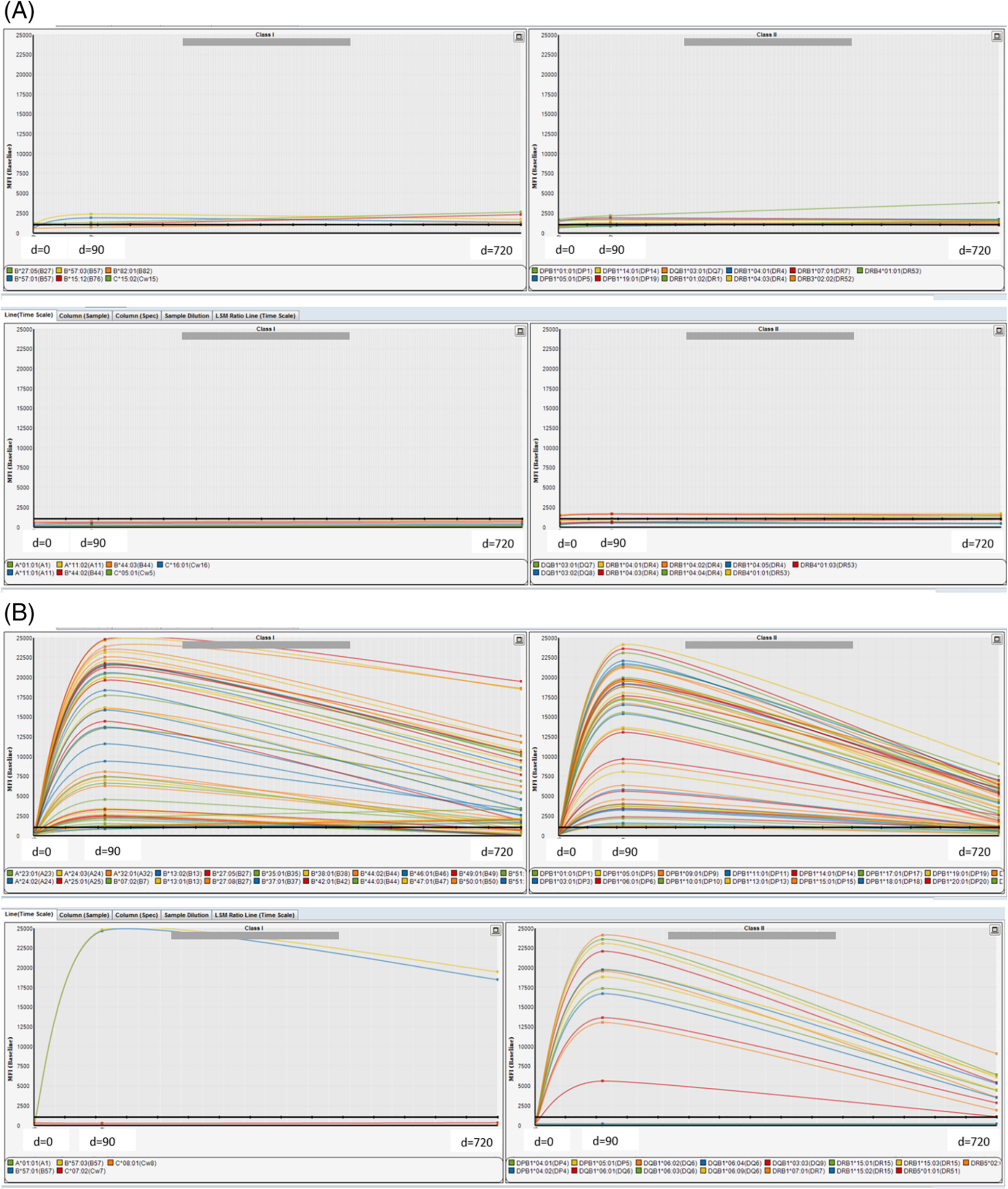
A, Tracking of HLA antibodies for class I (left column) and class II (right column) at d = 0, d = 90, and d = 720 of each one patient receiving a decellularized homograft. The x‐axis shows the time course, where d = 0 denotes the day of implantation, d = 90 and d = 720 denotes the days of the measurements of HLA‐specific antibodies after implantation; the y‐axis indicates MFI values as a measure of the amount of HLA‐specific antibodies (MFI max = 25 000). Time course of the MFI levels has been interpolated by the Fusion software. Each colored line represents the course of an antibody with defined HLA‐specificity (indicated in the lowest part of each plot). The horizontal black bold line indicates an MFI of 1000. Antibody profiles for a patient with a fresh decellularized homograft are shown. The upper lane shows all detected HLA Class I or HLA Class II antibodies with an MFI > 1000. The lower lane shows donor‐specific HLA Class I (DSA Class I) or HLA Class II (DSA Class II) antibodies. They are directed to HLA antigens of the donor of the homograft. B, Tracking of HLA antibodies for class I (left column) and class II (right column) at d = 0, d = 90, and d = 720 of each one patient receiving a cryopreserved native homograft. The x‐axis shows the time course, where d = 0 denotes the day of implantation, d = 90 and d = 720 denotes the days of the measurements of HLA‐specific antibodies after implantation; the y‐axis indicates MFI values as a measure of the amount of HLA‐specific antibodies (MFI max = 25 000). Time course of the MFI levels has been interpolated by the Fusion software. Each colored line represents the course of an antibody with defined HLA‐specificity (indicated in the lowest part of each plot). The horizontal black bold line indicates an MFI of 1000. Antibody profiles for patients with cryopreserved native homograft are shown. The upper lane shows all detected HLA Class I or HLA Class II antibodies with an MFI > 1000. The lower lane shows donor specific HLA Class I (DSA Class I) or HLA Class II (DSA Class II) antibodies. They are directed to HLA antigens of the donor of the homograft

The tracking of antibodies from pre‐ (d = 0) to post‐homograft implantation (d = 90 and d = 720) can be indicated with the Fusion Software (Thermo Fisher Scientific Inc.). The tracking of one patient of each homograft group is shown in Figure 2 (Figure 2A indicates a patient receiving a fresh decellularized homograft and Figure 2B a patient implanted with a native cryopreserved homograft). This graphical representation indicates the broad immunization against allogeneic HLA antigens. The MFI of both, global allogeneic HLA antibodies and donor‐specific antibodies were significantly higher in patients receiving native cryopreserved allografts.

Supplemental Table 2 summarizes all HLA typings of the donors and the characteristics of donor specific antibodies.

Information about ABO typing was available only for the decellularized group as we obtained the homografts from patients undergoing heart transplantation at our institution. Only one patient was ABO compatible with the homograft donor; the antibodies levels were not significantly different at 3‐months for ABO compatibility vs incompatibility.

### 3.4 Hemodynamic and clinical outcome

There was no operative or late mortality. No major postoperative complications related to homograft implantation occurred. The mean gradient at 2‐years follow‐up for the cryopreserved native homograft group (all in pulmonary position) was 6 ± 2 mmHg and 11 mmHg for one patient receiving a decellularized pulmonary homograft; the other three patients from the decellularized group received an aortic homograft and showed a mean gradient of the aortic valve at 2 years of 12 ± 8 mmHg. At the last 2‐years follow‐up, functional New York Heart Association classification was I for all patients.

## 4 DISCUSSION

Cryopreserved homografts were extensively used in the treatment of different congenital defects implying the pulmonary valve/artery or acquired aortic stenosis/insufficiency in children or young adults, either as isolated pulmonary root replacement, the Ross operation, or isolated aortic root replacement. Cryopreservation itself permits long‐term tissue storage without altering the integrity of the tissue. The preservation of endothelium viability increases the immunogenicity of the allografts and enhances the immune response of recipients. Our analysis demonstrated a vigorous immune response with increased HLA class I and class II antibodies after cryopreserved homograft implantation and a minimal/absent immunological response after fresh decellularized homograft implantation. Three months after implantation the group of patients having received the cryopreserved allografts showed significantly higher sums of MFIs (*P* = .021) as compared with the patients receiving decellularized allografts. This indicates that a broad immunization against allogeneic HLA antigens has taken place in the native cryopreserved group while patients having received decellularized antibodies showed no or minimal immunization only. We were able to detect several different types of HLA antibodies induced by the homograft implantation, even more, intensive compared with the reaction after a transplantation because of the absent immunosuppressive therapy. The implication of this immune response has to be taken into consideration especially in children with complex congenital defects in whom later heart transplantation might be required. These patients have undergone multiple operations, often with the use of allograft pledget material or homografts and received multiple blood transfusions which also predispose to HLA antibody formation. The fact that the class II antibodies are present is, at a first glance, startling, since class II antigens are not constitutively expressed on the transplanted tissue. Inflammatory process, however, could induce the class II antigen expression which could in turn induce an HLA class II specific antibody response. It might also be, that some immunologically active class II positive cells, for example, dentritic cells are present in the transplanted tissue.

The presence of HLA antibodies was shown to increase the risk of acute or hyperacute graft rejection in these patients. HLA antibodies elevation requires cross‐matching before heart transplantation and decreases the number of possible matching donors. All four patients receiving a native cryopreserved homograft showed >95% vPRAs. Therefore, if they would require a transplantation, most donors would be unsuitable for these patients. We previously reported a case of acute rejection in patients who underwent a Ross procedure with a cryopreserved homograft.^8^

Breinholt et al^9^ analyzed the level of HLA class I and class II panel reactive antibodies (PRAs) after implantation of cryopreserved allograft material for the repair or palliation of congenital heart disease in 11 patients. Within 3 months of implantation, class I and class II panel reactive antibody levels, determined by flow‐cytometry analysis, increased to 70 ± 38% and 41 ± 36%, when compared with baseline. Hawkins et al^10^ analyzed the immunogenicity after implantation of decellularized cryopreserved allografts in 14 children and compared the results with a control population implanted with standard native cryopreserved allografts. They observed significantly lower levels of class I and class II HLA antibody formation at 1, 3, and 12 months after implantation of decellularized cryopreserved allografts than with standard cryopreserved grafts. A slight elevation of both class I and class II HLA antibodies was observed from baseline at patients receiving a decellularized allograft at 1, 3, and 12 months but the values were not significant. Da Costa et al. compared the immunological and echocardiographic data between 11 patients receiving a decellularized pulmonary homograft and 9 patients implanted with a standard cryopreserved homograft up to 6 months post‐Ross procedure.^11^ Cryopreserved homografts showed marked elevations for class I and class II HLA antibodies which started at the first month and remained increased at 6 months, in contrast with the decellularized group, in which 9 patients had an absent or very mild immune response and two patients, one of them with increased antibodies preoperatively and the second only with a transitory early postoperative immunoreactivity which normalized at 6 months.

The increased immunogenicity expressed by HLA antibodies class I and class II, as well as ABO mismatch were associated with accelerated homograft valve failure in the pediatric population.^12^

Kneib et al^5^ studied a total of 12 patients with cellularized and decellularized allografts. He found a higher degree of donor‐specific antibodies on the level of mismatched epitopes. We also studied the occurrence of DSA and, similarly, found a higher degree of DSA in the group receiving cryopreserved native implants. However, the amount of the DSA is dependent on the number of mismatches alleles or epitopes. This number might vary statistically from case to case. For this reason, we also look at the general degree of immunization and found that in all four patients with cryopreserved native homografts a very strong immunization took place: the antibodies were directed against many different specificities. This might be explained by shared epitopes between these specificities. However, more clinically important, this broad range of allogeneic antibody specificities might be a considerable immunological risk factor for future transplantations. This risk affects not only heart transplantation, but also the transplantation of other solid organs or stem cells.

Oeser et al^3^ analyzed 274 patients receiving a pulmonary homograft at our institution between 1991 and 2014 revealed that homografts obtained from the Vienna Homograft Bank between 1991 and 2008, showed a significant higher rate of freedom of reintervention compared with homografts obtained from Cryolife Inc. Homografts from Vienna Homograft Bank were harvested only from heart‐beating donors, were fresh native preserved and implanted between 1991 and 2008 in this series of patients; contrary, the homografts from Cryolife were obtained from non‐heart‐beating donors. Our homograft harvesting facility started again in 2015 along with the possibility to send the fresh homografts to decellularization at Corlife, which were returned fresh at our institution for implantation, as we described above. The different donor type as well as the preservation method and the decellularization process may play a role in the later structural degeneration process.

The fresh decellularized grafts analyzed in our cohort are currently approved and available in Europe and investigated in two big, EU‐funded projects and will be further implanted in several centers. The specific aspect of these grafts compared with other decellularized grafts is that they are not frozen, which may not influence immunogenicity but may significantly improve long‐term durability, increasing the likelihood of a successful therapeutic approach. Another unique aspect of this study was the possibility to perform the HLA typing from the valve tissue, even if the donor is not or no longer available. This information might be important in case of future transplantation either with solid organs or with hematopoietic stem cells.

Boethig et al analyzed 235 patients implanted with a decellularized pulmonary homograft included in the prospective multicenter trial ESPOIR and compared the results with two different control groups—patients receiving either a standard cryopreserved pulmonary homograft or a bovine jugular vein graft in pulmonary position.^6^ At 10‐years follow‐up, the decellularized pulmonary homograft group showed significantly better freedom from valve explantation compared with standard cryopreserved pulmonary homografts (*P* = .029) or bovine jugular vein grafts (*P* = .012) and less structural valve degeneration when matched to bovine jugular vein conduits (*P* = .029), but no significant difference to standard cryopreserved pulmonary homograft. Sarikouch et al^13^ showed in a recent study important in vivo recellularization with noninflammatory cells in 11 of 364 patients implanted either with an aortic or pulmonary decellularized homograft between 2010 and 2017 in whom a reoperation with graft explanation was necessary for different reasons. The initial results of the multicentric European trial ARISE, analyzing this particular type of fresh decellularized homografts implanted in aortic position in 144 patients also demonstrated excellent early hemodynamic results.^14^

### 4.1 Study limitations

The small number of patients included and the limited follow‐up to 2 years is insufficient to observe any differences in possible earlier graft failure in the native cryopreserved group. A long‐term prospective clinical and immunological analysis of both native cryopreserved and fresh decellularized homografts has to be conducted.

In conclusion, almost no stimulation of class I and class II HLA antibodies was observed after implantation of fresh decellularized allografts in comparison with the cryopreserved native allografts. Lower immunogenicity will probably improve graft function and reduce the risk of rejection if transplantation may be required later in life.

#### Conflict of interests

The authors have declared no conflicting interests.

## DISCLOSURES

This study was conducted with institutional resources. No external funding was used. All authors have read and approved the submission of the manuscript. All authors have contributed significantly to the content of the article. Subject to acceptance, authors will sign an exclusive license to publish.

## Supporting information


**AppendixS1**: supporting information

## Data Availability

The data that support the findings of this study are available on request from the corresponding author. The data are not publicly available due to privacy or ethical restrictions.
