# Supplementary material for: Donor‐specific HLA antibodies after fresh decellularized vs cryopreserved native allograft implantation
Source: HLA. 2020 Oct 13;96(5):580–8. doi: 10.1111/tan.14077 (PMC7702054; doi:10.1111/tan.14077)
Supplement: Supplementary file 1 — AppendixS1: supporting information [file TAN-96-580-s001.docx]

**Supplemental Table 1**

Supplemental Table 1. Legend: The table shows the sum of MFI values of all HLA specific antibodies for each patient divided into HLA class I and class II specific antibodies for d=0 and d=90

| Group | Patient | Class I_d=0 | Class I_d=90 | Class II_d=0 | Class II_d=90 |
| --- | --- | --- | --- | --- | --- |
| Cryo | 1 | 14 281 | 640 726 | 11 990 | 654 686 |
|  | 2 | 14 905 | 887 052 | 15 210 | 102 265 |
|  | 3 | 13 335 | 1 010 558 | 11 218 | 242 581 |
|  | 4 | 21 940 | 844 911 | 25 864 | 371 489 |
| Decel | 1 | 24 809 | 35 765 | 29 001 | 39 754 |
|  | 2 | 23 106 | 22 302 | 21 141 | 21 972 |
|  | 3 | 7 379 | 25 094 | 13 490 | 43 699 |
|  | 4 | 24 323 | 18 899 | 17 284 | 12 874 |

Supplemental Table 2 shows the results of HLA typing of donors and donor specific antibodies. Donors are characterized by their HLA typings at high or low resolution. The HLA type of the cryopreserved valves had been typed historically, because they were taken from the hearts of patients receiving a new organ. These typings had been performed at low resolution. In the table we show the MFI values of beads carrying HLA molecules that belong to the same antigen.

The HLA type of the decellularized valves had been typed from the tissue. The typing was performed by NGS at the high resolution level.

MFI values of beads carrying recombinant HLA molecules that correspond to the donor are shown for all patients on day 0 and day 90.

If only low-resolution typing of donors was available we show the values of the beads carrying the HLA molecules matched at two fields. In the case of a missing HLA molecule on the beads (e.g. there is no bead in the test that carries C*07:01) the value of all beads carrying the corresponding antigen is shown.

Supplemental Table 2 summarizes all HLA typings of the donors and the characteristics of donor specific antibodies

**Supplemental Table 2**. Donor Typing and Donor Specific Antibody Characteristics

| A*01:01,B*14:02,B*57:01,C*07:01,C*08:02,DRB1*07:01,DRB1*15:01,DRB4*01:03N,DRB5*01:01,DQA1*01:02,DQA1*02:01,DQB1*03:03,DQB1*06:02,DPB1*04:01,DPB1*05:01 | | | | | | | | | | | | | | | |
| --- | --- | --- | --- | --- | --- | --- | --- | --- | --- | --- | --- | --- | --- | --- | --- |
| A*01:01 | B*57:01 | B*14:02 | C*07:02 | C*08:01 |  |  |  |  |  |  |  |  |  |  |  |
| 56 | 129 | 209 | 299 | 108 |  |  |  |  |  |  |  |  |  |  |  |
| 0 | 24665 | 73 | 206 | 36 |  |  |  |  |  |  |  |  |  |  |  |
| DPB1*04:01 | DPB1*05:01 | DQB1*06:02 | DQB1*03:03 | DRB1*07:01 | DRB1*15:01 | DRB5*01:01 |  |  |  |  |  |  |  |  |  |
| 155 | 194 | 158 | 215 | 199 | 153 | 289 |  |  |  |  |  |  |  |  |  |
| 148 | 19663 | 19512 | 5608 | 24094 | 17347 | 13632 |  |  |  |  |  |  |  |  |  |
| A*02:01,B*27:05,B*40:01,C*01:02,C*03:04,DRB1*01:01,DRB1*13:02,DRB3*03:01,DQA1*01:01,DQA1*01:02,DQB1*05:01,DQB1*06:09,DPB1*04:01,DPB1*138:01 | | | | | | | | | | | | | | | |
| A*02:01 | B*27:05 | B*40:01 | C*01:02 | C*03:02 |  |  |  |  |  |  |  |  |  |  |  |
| 70 | 82 | 143 | 325 | 436 |  |  |  |  |  |  |  |  |  |  |  |
| 25807 | 23811 | 19318 | 18941 | 20089 |  |  |  |  |  |  |  |  |  |  |  |
| DPB1*04:01 | DPB1*23:01 | DQB1*05:01 | DQB1*06:09 | DRB1*01:01 | DRB1*13:01 | DRB1*13:03 | DRB3*03:01 |  |  |  |  |  |  |  |  |
| 106 | 90 | 77 | 66 | 54 | 79 | 102 | 111 |  |  |  |  |  |  |  |  |
| 149 | 137 | 270 | 169 | 7161 | 3949 | 3920 | 8110 |  |  |  |  |  |  |  |  |
| A*01:01,A*03:01,B*35:01,B*57:01,C*04:01,C*06:02,DRB1*01:01,DRB1*04:01,DRB4*01:03,DQA1*01:01,DQA1*03:03,DQB1*03:01,DQB1*05:01,DPB1*04:01 | | | | | | | | | | | | | | | |
| A*01:01 | A*03:01 | B*35:01 | B*57:01 | C*04:01 | C*06:02 |  |  |  |  |  |  |  |  |  |  |
| 97 | 40 | 109 | 73 | 348 | 180 |  |  |  |  |  |  |  |  |  |  |
| 23178 | 22059 | 23169 | 23175 | 4419 | 7214 |  |  |  |  |  |  |  |  |  |  |
| DPB1*04:01 | DQB1*05:01 | DQB1*03:01 | DRB1*01:01 | DRB1*04:01 | DRB4*01:03 |  |  |  |  |  |  |  |  |  |  |
| 128 | 73 | 122 | 94 | 126 | 245 |  |  |  |  |  |  |  |  |  |  |
| 2985 | 908 | 7056 | 22495 | 14673 | 252 |  |  |  |  |  |  |  |  |  |  |
| A*02:01,A*24:02,B*07:02,B*51:01,C*07:02,C*15:02,DRB1*01:03,DRB1*03:01,DRB3*02:02,DQA1*01:01,DQA1*05:01,DQB1*02:01,DQB1*05:01,DPB1*02:01 | | | | | | | | | | | | | | | |
| A*02:01 | A*24:02 | B*07:02 | B*51:01 | C*07:02 | C*15:02 |  |  |  |  |  |  |  |  |  |  |
| 39 | 213 | 82 | 152 | 379 | 271 |  |  |  |  |  |  |  |  |  |  |
| 0 | 19740 | 21271 | 24016 | 215 | 15845 |  |  |  |  |  |  |  |  |  |  |
| DPB1*02:01 | DQB1*02:01 | DQB1*05:01 | DRB1*01:01 | DRB1*01:02 | DRB1*03:01 | DRB3*02:02 |  |  |  |  |  |  |  |  |  |
| 201 | 187 | 319 | 207 | 508 | 314 | 210 |  |  |  |  |  |  |  |  |  |
| 13396 | 174 | 20152 | 13253 | 12147 | 311 | 5124 |  |  |  |  |  |  |  |  |  |
| A*01,A*11,B*44,C*05,C*16,DRB1*04:01,DRB1*04:02,DQB1*03 | | | | | | | | |  |  |  |  |  |  |  |
| A*01:01 | A*11:01 | A*11:02 | B*44:02 | B*44:03 | C*05:01 | C*16:01 |  |  |  |  |  |  |  |  |  |
| 26 | 95 | 0 | 521 | 701 | 88 | 218 |  |  |  |  |  |  |  |  |  |
| 96 | 188 | 43 | 587 | 703 | 242 | 398 |  |  |  |  |  |  |  |  |  |
| DQB1*03:01 | DQB1*03:02 | DRB1*04:01 | DRB1*04:02 | DRB1*04:04 | DRB1*04:05 | DRB1*04:03 | DRB4*01:01 | DRB4*01:03 |  |  |  |  |  |  |  |
| 960 | 309 | 1533 | 772 | 326 | 393 | 1404 | 747 | 513 |  |  |  |  |  |  |  |
| 1122 | 495 | 1699 | 964 | 587 | 540 | 1627 | 904 | 601 |  |  |  |  |  |  |  |
| A*02:01,A*03:01,B*40:01,B*51:01,C*02:02,C*03:04,DRB1*11:01,DRB1*13:02,DRB3*02:02,DRB3*03:01,DQA1*01:02,DQA1*05:05,DQB1*03:01,DQB1*06:04,DPB1*03:01,DPB1*04:01 | | | | | | | | | | | | | | | |
| A*02:01 | A*03:01 | B*51:01 | B*40:01 | C*02:02 | C*03:04 |  |  |  |  |  |  |  |  |  |  |
| 82 | 71 | 165 | 240 | 344 | 359 |  |  |  |  |  |  |  |  |  |  |
| 55 | 41 | 99 | 228 | 304 | 306 |  |  |  |  |  |  |  |  |  |  |
| DPB1*03:01 | DPB1*04:01 | DQB1*06:04 | DQB1*03:01 | DRB1*11:01 | DRB1*13:01 | DRB1*13:03 | DRB3*03:01 |  |  |  |  |  |  |  |  |
| 247 | 179 | 209 | 250 | 281 | 160 | 152 | 211 |  |  |  |  |  |  |  |  |
| 287 | 156 | 198 | 314 | 277 | 136 | 132 | 251 |  |  |  |  |  |  |  |  |
| A*02,A*26,B*08,B*56,C*01,C*07,DRB1*03:01,DRB1*15:01,DQB1*02,DQB1*06 | | | | | | | | | | |  |  |  |  |  |
| A*02:01 | A*02:03 | A*02:06 | A*26:01 | B*08:01 | B*56:01 | C*01:02 | C*07:02 |  |  |  |  |  |  |  |  |
| 0 | 0 | 0 | 110 | 0 | 0 | 223 | 55 |  |  |  |  |  |  |  |  |
| 0 | 0 | 17 | 256 | 18 | 150 | 518 | 217 |  |  |  |  |  |  |  |  |
| DQB1*02:01 | DQB1*02:02 | DQB1*06:01 | DQB1*06:02 | DQB1*06:03 | DQB1*06:04 | DQB1*06:09 | DRB1*15:01 | DRB1*15:02 | DRB1*15:03 | DRB1*03:01 | DRB5*01:01 | DRB5*02:02 | DRB3*01:01 | DRB3*02:02 | DRB3*03:01 |
| 12 | 33 | 2536 | 1502 | 3874 | 692 | 2083 | 60 | 49 | 0 | 0 | 8 | 0 | 94 | 46 | 68 |
| 198 | 152 | 5807 | 4189 | 8220 | 2705 | 5381 | 356 | 375 | 240 | 193 | 501 | 204 | 392 | 365 | 274 |
| A*02,A*31,B*18,B*51,C*07,C*14,DRB1*04:03,DRB1*08:01,DQA1*03:01,DQA1*04:02,DQB1*03:02,DQB1*04:02,DPB1*03:01,DPB1*10:01 | | | | | | | | | | | | | | | |
| A*02:01 | A*02:03 | A*02:06 | A*31:01 | B*18:01 | B*51:01 | B*51:02 | C*07:02 | C*14:02 |  |  |  |  |  |  |  |
| 131 | 83 | 96 | 125 | 131 | 232 | 211 | 287 | 253 |  |  |  |  |  |  |  |
| 58 | 27 | 48 | 84 | 42 | 161 | 132 | 217 | 222 |  |  |  |  |  |  |  |
| DPB1*03:01 | DPB1*10:01 | DQB1*04:01 | DQB1*04:02 | DQB1*03:02 | DRB1*04:01 | DRB1*04:02 | DRB1*04:04 | DRB1*04:05 | DRB1*04:03 | DRB1*08:01 | DRB4*01:01 | DRB4*01:03 |  |  |  |
| 174 | 204 | 322 | 278 | 170 | 757 | 379 | 186 | 301 | 691 | 201 | 262 | 249 |  |  |  |
| 106 | 153 | 233 | 247 | 140 | 664 | 312 | 109 | 228 | 621 | 142 | 178 | 183 |  |  |  |
